# Impacts of Commonly Used Edible Plants on the Modulation of Platelet Function

**DOI:** 10.3390/ijms23020605

**Published:** 2022-01-06

**Authors:** Dina A. I. Albadawi, Divyashree Ravishankar, Thomas M. Vallance, Ketan Patel, Helen M. I. Osborn, Sakthivel Vaiyapuri

**Affiliations:** 1 School of Pharmacy, University of Reading, Reading RG6 6UB, UK; d.a.i.albadawi@pgr.reading.ac.uk (D.A.I.A.); divyasri.april86@gmail.com (D.R.); tmv31@cam.ac.uk (T.M.V.); 2 School of Biological Sciences, University of Reading, Reading RG6 6UB, UK; ketan.patel@reading.ac.uk

**Keywords:** platelets, aggregation, cardiovascular diseases, atherosclerosis, fruits, vegetables, spices

## Abstract

Cardiovascular diseases (CVDs) are a primary cause of deaths worldwide. Thrombotic diseases, specifically stroke and coronary heart diseases, account for around 85% of CVDs-induced deaths. Platelets (small circulating blood cells) are responsible for the prevention of excessive bleeding upon vascular injury, through blood clotting (haemostasis). However, unnecessary activation of platelets under pathological conditions, such as upon the rupture of atherosclerotic plaques, results in thrombus formation (thrombosis), which can cause life threatening conditions such as stroke or heart attack. Therefore, antiplatelet medications are usually prescribed for people who are at a high risk of thrombotic diseases. The currently used antiplatelet drugs are associated with major side effects such as excessive bleeding, and some patients are resistant to these drugs. Therefore, numerous studies have been conducted to develop new antiplatelet agents and notably, to establish the relationship between edible plants, specifically fruits, vegetables and spices, and cardiovascular health. Indeed, healthy and balanced diets have proven to be effective for the prevention of CVDs in diverse settings. A high intake of fruits and vegetables in regular diet is associated with lower risks for stroke and coronary heart diseases because of their plethora of phytochemical constituents. In this review, we discuss the impacts of commonly used selected edible plants (specifically vegetables, fruits and spices) and/or their isolated compounds on the modulation of platelet function, haemostasis and thrombosis.

## 1. Introduction

According to the World Health Organisation, cardiovascular diseases (CVDs) are a major cause of deaths worldwide [1]. In 2017, more than 85% of total CVDs-associated deaths were caused by thrombotic diseases (primarily strokes and coronary heart diseases), and they are largely triggered by the rupture of atherosclerotic plaques [1,2]. Numerous studies have demonstrated that the effective management of risk factors for CVDs is critical for their prevention and management, as well as to improve the quality of life for patients in the long-term [3]. An unhealthy diet is a key risk factor for CVDs, as the regular consumption of food which has high levels of saturated fats, sugar, sodium, animal proteins and low in fibre (fruit and vegetables) can cause hypertension, hyperglycaemia, hyperlipidaemia and obesity, which ultimately result in CVDs [4,5,6].

CVDs are a group of pathological conditions that affect the heart and blood vessels due to both non-modifiable and modifiable risk factors. Non-modifiable risk factors include age, gender, ethnicity, and family history for CVDs. However, modifiable risk factors involve high blood pressure, increased cholesterol level, high blood glucose, unhealthy diet, obesity, reduced physical activities, smoking and stress [7]. The most common type of CVDs, coronary artery disease, is mainly caused by the formation/rupture of atherosclerotic plaques within coronary arteries leading to reduced blood supply to heart muscles [7]. Atherosclerotic plaques are formed due to the accumulation of low-density lipoproteins (LDL) in the arterial intima, which results in endothelial dysfunction, initiation of inflammatory responses and the formation of oxidised LDL [8]. As a result, immune cells such as monocytes are attracted to the damaged site by chemokines, and inflammatory markers are released from the affected region. The migrated monocytes differentiate into macrophages that engulf oxidised LDL and become foam cells [8,9]. The accumulation of an excessive amount of lipids over time causes necrosis, fibrous tissue formation and calcification which increases the plaque size and subsequently leads to a reduction in blood flow inside the affected arteries [7]. Notably, the plaque can rupture and trigger the formation of blood clots (thrombi) within the blood vessels. Thrombosis can restrict the blood flow to vital organs such as the heart and brain, and can cause myocardial infarction or ischemic stroke, respectively [10].

At the site of vascular damage, the subendothelial matrix and its contents, mainly collagen, are exposed to circulation. Collagen adheres to circulating platelets by binding to von Willebrand factor (vWF) (immobilised on fibrillar collagen type I and III), platelet glycoprotein (GP) Ib-V-IX receptor complex and GPVI receptor and facilitates their activation at the injury site [11]. As a result, a monolayer of platelets is formed to cover the damaged region. Notably, GPVI plays an essential role in collagen-mediated platelet activation, and also promotes a stable adhesion of collagen via integrin α2β1. GPVI-mediated signalling activates phosphoinositol-3-kinase (PI3K) and phospholipase Cγ2 leading to intracellular calcium mobilisation, inside-out signalling to integrin αIIbβ3, granule secretion and finally, platelet aggregate or thrombus formation [11,12]. In addition to collagen-mediated activation, more platelets are activated by autocrine and paracrine signalling through adenosine diphosphate (ADP) (released from platelet dense granules) and thromboxane A_2_ (TXA_2_, synthesised and released from activated platelets). Fibrinogen (present in plasma) is the major ligand for integrin αIIbβ3 on the surface of activated platelets and it promotes platelet aggregation by acting as a bridge between platelets [13]. In addition, thrombin is produced as a result of coagulation cascades and it activates more platelets via protease-activated receptors (PARs) and converts fibrinogen into fibrin to form a polymerised fibrin network, resulting in a stable clot/thrombus formation [14,15].

Platelets are small, anucleated circulating blood cells derived from megakaryocytes in the bone marrow. They have a life span of around 8–10 days and their normal count in blood is between 150–400 × 10^9^/L. The primary function of platelets is to avoid excessive blood loss upon vascular injury by forming a blood clot under physiological conditions (this process is commonly known as ‘haemostasis’) [11]. However, as detailed above, platelets become unnecessarily activated under various pathological conditions, leading to thrombosis [11,16]. Therefore, antiplatelet medications are predominantly prescribed for the primary and secondary prevention of thrombotic diseases [17,18]. The most commonly prescribed antiplatelet drugs are aspirin and clopidogrel either as monotherapy (single drug) or dual therapy (two drugs) to achieve their optimal/maximal effects [19,20]. However, even dual antiplatelet treatments may not prevent the recurrence of thrombotic incidents in some patients and many patients develop resistance to aspirin and/or clopidogrel due to a number of reasons, including inadequate dosing, drug–drug interactions and poor patient compliance [21,22]. Moreover, excessive bleeding is a major side effect of these medications and may lead to the need for blood transfusion in some cases. The bioavailability of clopidogrel may be affected by co-administered drugs that are metabolised in the liver by CYP2C19 and CYP3A4/5 enzymes and this may affect the decision on prescribing it for some patients [22].

Notably, an unhealthy diet that consists of high sugar, sodium and saturated fats and lower amounts of fruit, vegetables, legumes, fibres, nuts and fish is a critical modifiable risk factor for several chronic diseases, specifically CVDs [23,24,25]. In most cases, a healthy and balanced diet is a part of the treatment/prevention plan for CVDs, including for thrombotic diseases [26]. Several studies have demonstrated the effects of plants-based diets in reducing risk factors for CVDs and the associated mortality rate [27]. In general, a daily intake of five servings of fruits/vegetables (plant-based foods) ranging from 400–800 g/day is recommended to reduce the risk for CVD [28]. The beneficial effects of fruits, vegetables and other plant-based foods are mainly related to their antioxidant, anti-inflammatory, hypotensive and hypoglycaemic effects as a result of a plethora of phytochemicals, including flavonoids, phenolic acids, alkaloids and glycosides as well as vitamins, minerals and fibres [29,30,31,32].

The outcomes of many observational studies demonstrated that a vegetarian diet has favourable effects on cardiovascular health to control obesity, hyperlipidaemia, hypertension and type II diabetes compared to non-vegetarian diets [33]. Vegetarians displayed lower body mass index (BMI), serum cholesterol level and diastolic blood pressure than non-vegetarians [33,34,35]. In addition, it is reported that vegetarian diets offer significantly lower risks for heart attacks and strokes [36,37,38]. Moreover, inflammatory biomarkers, including interleukin-6 (IL-6), C-reactive protein (CRP) and tumour necrosis factor-alpha (TNF-α) were significantly lower among vegetarians [39,40].

Overall, although antiplatelet drugs are the first line of defence for the prevention and treatment of CVDs (specifically thrombotic diseases), they are associated with serious side effects. Therefore, there is an urgent need to develop novel antiplatelet agents that are more effective, safer, and affordable for short and long-term management of thrombotic diseases/CVDs. Accordingly, numerous studies have investigated the impacts of various edible plants and/or their isolated active compounds on the modulation of platelet function. In this review, the effects of various vegetables, fruits, spices, and edible fungi and their isolated compounds in the modulation of platelet activation and thrombosis are discussed. Whilst some reviews were published on a specific plant compound or a group of compounds, specific plants and their effects on platelets, this review will focus on the effects of both plant extracts and their isolated compounds on the modulation of platelet activation under diverse settings in an integrated manner.

## 2. Vegetables

### 2.1. Onions

*Allium cepa* (onion) is one of the main *Allium* spices that were studied for their beneficial effects on human health, specifically on the cardiovascular system. Their antioxidant, antihypertensive, antiplatelet, anti-inflammatory and antihyperlipidemic effects were analysed in various settings [41,42]. Onions are a widely cultivated and consumed vegetable all over the world, although they originate from Central Asia. Traditionally, onions were used to treat cold, flu, dysentery, wound healing, and alleviate pain [42]. Several studies have explored the different classes of phytochemicals present in *A. cepa* including volatile oil, and sulphur-containing compounds such as methyl 5-methylfuryl sulphide and dimethyl disulphide which are responsible for their characteristic flavour. In addition, phenolic compounds such as phenolic acids (e.g., *p*-hydroxybenzoic acid and gallic acid) and flavonoids (e.g., anthocyanins, quercetin and kaempferol) were isolated and characterised from onions [43,44,45]. These active constituents are associated with the biological effects of *A. cepa*, including antioxidant, antibiotic, anti-cancer, anti-diabetic, anti-inflammatory and anti-allergic activities. In addition, they significantly reduce CVD risks through hypolipidemic, hypotensive, hypoglycaemic and antiplatelet effects [41,42].

**Figure 1:**
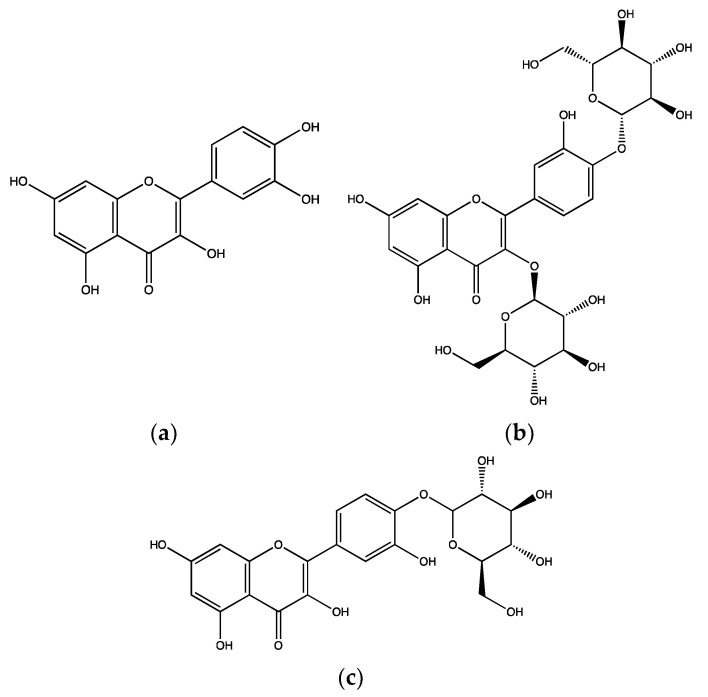
Chemical structures of quercetin (**a**)**,** quercetin-3,4′-*O*-diglucoside (**b**) and quercetin-4′-*O*-monoglucoside (**c**) isolated from *Allium cepa*.

To evaluate the antiplatelet effects of *A. cepa* bulb, different concentrations of its aqueous extracts (250 and 500 mg/mL) were tested in human isolated platelets using an aggregation assay upon stimulation with a TXA_2_ receptor agonist, U46619 (2 µM). Both concentrations of the extract (250 and 500 mg/mL) significantly inhibited platelet aggregation by around 85–100% [46]. In addition, an ethanolic extract of *A. cepa* bulb showed significant antiplatelet effects when 5 µg/mL collagen was used as an agonist in rat isolated platelets through reducing intracellular Ca^2+^ levels, cyclooxygenase 1 enzyme (COX-1) and TXA_2_ synthase activities. It also increased cAMP levels in a concentration dependent manner [47]. The anti-aggregatory effects of *A. cepa* are attributable to the abundant flavonoid, quercetin (Figure 1a) and its glycosides, quercetin-3,4′-*O*-diglucoside (Figure 1b) and quercetin-4′-*O*-monoglucoside (Figure 1c). These compounds were isolated from the methanolic extract of *A. cepa* and at a concentration of 2 mg/mL, they completely inhibited 6 µg/mL collagen-induced platelet (rat) aggregation [48].

Furthermore, to test the impact of cooking methods and cooking time on *A. cepa* bulb-mediated antiplatelet effects, conventional (200 °C) and microwave (500 W, which is almost equivalent to 200 °C) ovens were selected to cook the samples for 10, 20 or 30 min. For conventional oven cooking, *A. cepa* samples were divided into; whole (intact) bulb, chopped into quarters, and crushed samples. For microwave cooking, only the whole bulbs and crushed samples were tested. Then, all samples were tested in human whole blood aggregation upon stimulation with 1 µg/mL collagen. First, a raw crushed sample of *A. cepa* was tested and it significantly inhibited platelet aggregation by around 85%. Samples that were cooked using the conventional oven showed different effects on platelet aggregation compared to the raw samples as shown in Table 1. These data suggested that the antiplatelet effects of *A. cepa* bulb can be lost due to aggressive processing using high temperature. In addition, the long cooking time can change the anti-aggregatory effects of *A. cepa*. On the other hand, samples that were cooked using the microwave method did not exert any inhibitory effects on platelets, irrespective of the cooking time [49].

**Figure 2:**
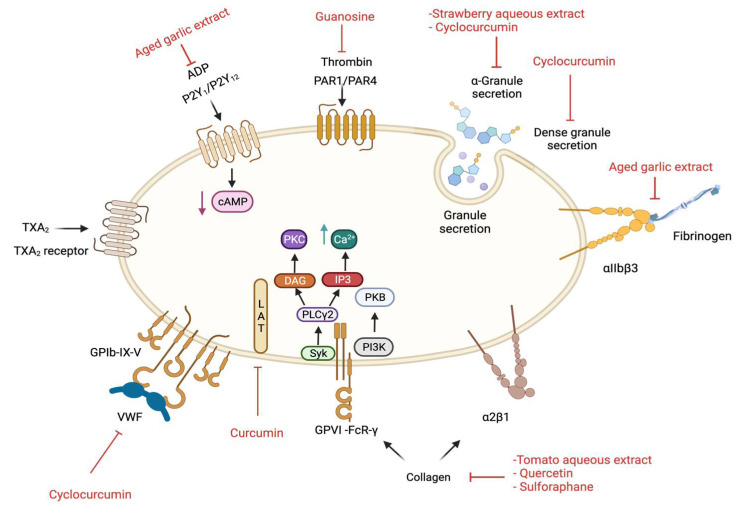
Signalling pathways that are affected by selective plant extracts and their compounds in platelets. cAMP; cyclic adenosine monophosphate, DAG; diacylglycerol, IP3; inositol trisphosphate, PI3K; phosphoinositide 3-kinase, PKB; Protein kinase B, PKC; Protein kinase C, PLCγ2; phospholipase Cγ2, and Syk; spleen tyrosine kinase. This image was created in BioRender.com (accessed on 23 December 2021). using the information provided in this article.

Furthermore, a human pilot study (*n* = 6) tested the acute effects of low (8.1 mg/L) and high (114.8 mg/L) quercetin (Figure 1a) contents in onion soups on human platelet activity via oral consumption. The anti-aggregatory effects of onion soups were evaluated in human isolated platelets upon activation with collagen (0.5, 1, 2 and 3 µg/mL) after 1 and 3 h of consumption. The soup with high quercetin content significantly inhibited platelet aggregation induced by different concentrations of collagen. In addition, the effect of both soups on tyrosine phosphorylation of spleen tyrosine kinase (Syk) and phospholipase C gamma 2 (PLCγ2) were evaluated, as they are crucial molecules in the signalling pathways of GPVI (a major collagen receptor). The high quercetin soup significantly inhibited the phosphorylation of Syk and PLCγ2 in 25 µg/mL collagen-induced platelets, when samples collected after 1 and 3 h of ingestion. In addition, the low quercetin soup insignificantly inhibited platelet aggregation induced by collagen while it stimulated tyrosine phosphorylation of Syk and PLCγ2 compared to the control at 1 and 3 h after ingestion (Figure 2) [50].

### 2.2. Garlic

**Figure 3:**
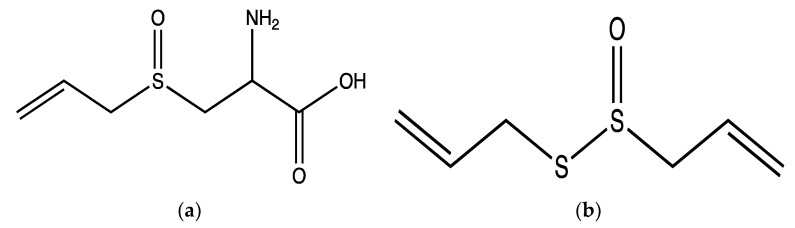
Structure of alliin (**a**) and (**b**) allicin isolated from the methanolic extract of *A. sativum*.

*Allium sativum* (garlic) is a common used vegetable and plays a critical role in the traditional medicine of many ancient cultures, such as the Egyptian, Indian, Chinese, Sumerian, and Greek. It was widely used to treat persistent cough, arthritis, constipation, snakebites and as a general antibiotic [51]. Various studies demonstrated that *A. sativum* exerts antioxidant, anti-inflammatory, anti-tumor, antibiotic, hypoglycaemic and renal protective effects [51,52]. In addition, it was reported that *A. sativum* has positive effects on CVDs through its antioxidant, hypotensive and hypocholesterolaemic effects. These biological effects are linked to sulphur-containing phytochemicals (including alliin and allicin) and enzymes (including alliinase and peroxidase) as well as flavonoids (e.g., quercetin) in *A. sativum* [52]. The antiplatelet effects of aqueous and methanolic extracts of *A. sativum* bulbs were examined in human PRP aggregation using different agonists; 20 µM ADP, 190 µg/mL collagen and 20 µM epinephrine. The aqueous extracts (10 mg/mL) significantly reduced ADP-induced aggregation by around 86% but did not affect aggregation induced by other agonists. However, the methanolic extracts (10 mg/mL) significantly inhibited ADP, epinephrin and collagen-induced aggregation by approximately 89%, 66% and 32%, respectively. The antiaggregatory effects of the methanolic extracts were suggested to be as a result of high contents of alliin (Figure 3a) and allicin (Figure 3b) in this plant [53]. However, the tested concentrations of agonists were higher than the commonly used concentrations (ADP: 0.5–10 µM; collagen: 1–5 µg/mL; epinephrin: 0.5–10 µM) for platelet aggregation [53]. Therefore, the inhibitory effects were not apparent.

Allicin is an organosulfur compound that accounts for around 70% of total thiosulfinates [contain the functional group, R-S(O)-S-R] in *A. sativum*. It is produced upon the physical disruption (e.g., by crushing or cutting) of tissues of *A. sativum* as the alliinase enzyme converts alliin to allicin upon damage [53]. Allicin and alliin are the main compounds responsible for the biological activities of *A. sativum* [52,54,55]. In human PRP and isolated platelets, the effect of 40 µM allicin was tested using 5 µg/mL collagen or 10 µM ADP and 55 µM epinephrine (combined)-induced platelet aggregation. In PRP, allicin did not affect aggregation, whilst in isolated platelets allicin markedly inhibited aggregation by around 98% in collagen and ADP-epinephrine activated platelets which indicates that the anti-aggregatory effects of allicin may be affected by plasma proteins in PRP. In addition, 40 µM allicin demonstrated significant inhibitory effects on fibrinogen binding and P-selectin exposure by around 80% and 90%, respectively, in ADP-epinephrine activated platelets [55]. However, in this study the combined usage of ADP-epinephrine as agonists was not justified. Moreover, the epinephrine concentration that was used appears to be higher than the commonly used concentrations (0.5–10 µM).

**Figure 4:**
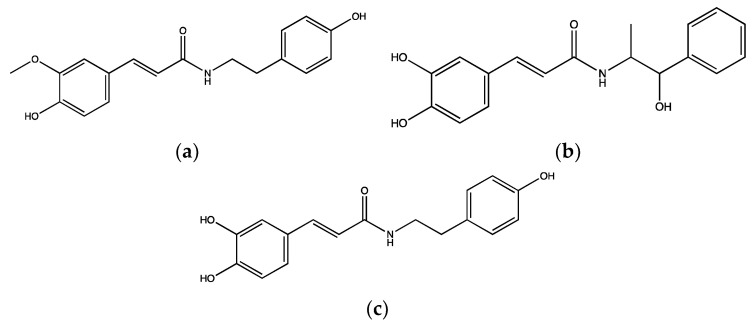
Chemical structures of (**a**) *N*-feruloyltyramine, (**b**) *N*-caffeoylnorephedrine and (**c**) *N*-caffeoyltyramine isolated from methanolic extracts of *A. sativum*.

In addition, *N*-feruloyltyramine (Figure 4a) is an amide alkaloid isolated from methanolic extracts of *A. sativum* and it is known to exhibit antioxidant, anti-fungal, anti-bacterial and cytotoxic effects [56]. This compound was tested in mouse whole blood along with its synthetic analogues, *N*-caffeoylnorephedrine (Figure 4b) and *N*-caffeoyltyramine (Figure 4c) to evaluate their effects on platelet function, specifically on COX-I enzyme and P-selectin exposure. *N*-feruloyltyramine and its analogues at the concentration of 0.05 µM significantly reduced the activity of COX-1. However, *N*-feruloyltyramine exhibited the highest inhibitory effect of around 43% compared to a COX-1 inhibitor, ibuprofen. In addition, *N*-feruloyltyramine, *N*-caffeoyltyramine and *N*-caffeoylnorephedrine (0.05 µM) significantly inhibited P-selectin exposure by 31%, 30% and 39%, respectively, in mouse whole blood upon stimulation with 2.5 µg/mL collagen [57].

Moreover, the extracts of *A. sativum* were evaluated after 20 months following their storage (aged extracts) for their antiplatelet effects. The aged extract was prepared by placing the chopped *A. sativum* bulb in ethanol or water/ethanol mixture at room temperature for 20 months. During this process, allicin, which is an unstable compound, is converted to a stable compound, namely *S*-allylcysteine. In addition, this extract has a higher content of total phenolic compounds compared to fresh *A. sativum* extracts (around 129 ± 1.8 mg/g compared to 56 ± 1.2 mg/g). Aged extracts showed better antioxidant, hypotensive, hypoglycaemic and hypolipidemic effects than fresh *A. sativum* extracts [58]. The aged extract prepared in 15–20% water: ethanol was tested in 8 µM ADP-induced human PRP aggregation at different concentrations; 0.29%, 0.58%, 1.56%, 3.12% and 6.25% (*v/v*). All these concentrations significantly inhibited platelet aggregation and fibrinogen binding (Figure 2) in a concentration dependant manner. Additionally, the extract markedly affected the change of platelet shape by blocking filopodia formation upon stimulation with ADP [59]. In another study, an aged extract [1.56%, 3.12%, 6.25%,12.5% and 25% (*v/v*)] significantly reduced human PRP aggregation induced by 8 µM ADP in a concentration dependant manner with almost 75% inhibition achieved at 25% (*v/v*) of the extract. In addition, 25% (*v/v*) extract significantly suppressed intracellular Ca^2+^ levels in 5 µM A23187 (a calcium ionophore) activated human isolated platelets [60]. Moreover, at concentrations of 3.12% and 12.5% (*v/v*), it markedly inhibited ADP-induced human PRP aggregation by around 40% by reducing the binding of platelets to fibrinogen and increasing the levels of intracellular cAMP (Figure 2) [61].

Furthermore, the antiplatelet effects of the aged extract were tested in rat PRP following oral administration. Three doses (1, 2 or 5 g/kg/day) of the extract were administered for 7 or 14 days in different cohorts of rats and then PRP was tested using 10 µg/mL collagen. All doses that were administered for 14 days significantly inhibited aggregation in a dose dependent manner. In isolated platelets obtained from rats treated with a 5 g/kg/day dose for 14 days, a significant inhibition of the phosphorylation of the mitogen-activated protein (MAP) kinases; p38, c-JUN NH2-terminal kinase (JNK) and extracellular signal-regulated kinase (ERK) (following activation with collagen), which are all important for platelets signalling was observed [62].

### 2.3. Wild Garlic

*Allium ursinum* has a less pungent taste than *A. sativum* (common garlic) and all of its parts are edible, although the leaves are typically consumed (raw or cooked) rather than the cloves, due to their high content of biologically active compounds, specifically organosulfur molecules [63]. The leaves were used in Asian, Middle Eastern and European folk medicine for their anti-bacterial, digestion stimulating and hypotensive effects [64]. Recent studies have demonstrated its antibiotic, cytotoxic, hypotensive, hypolipidemic and antiplatelet effects [63].

**Figure 5:**
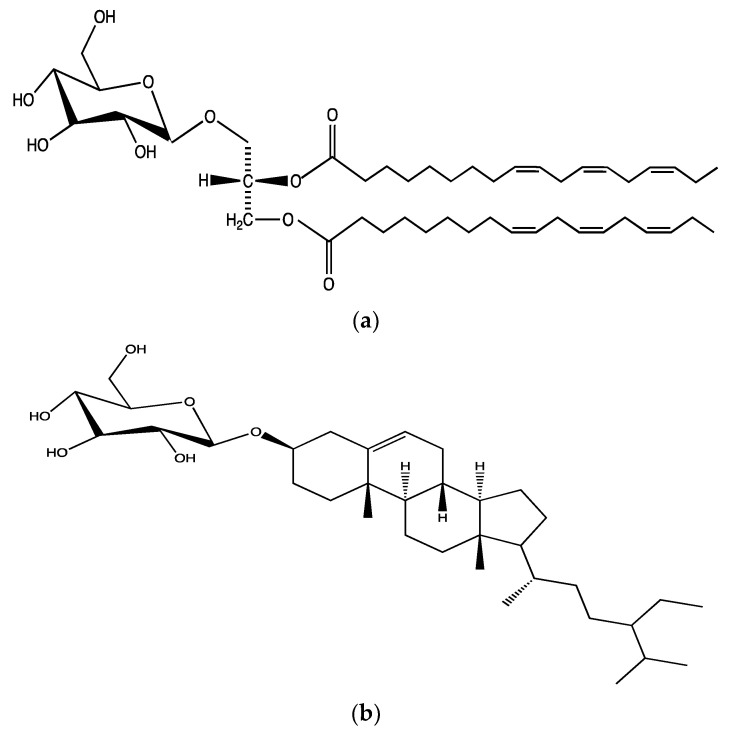
Chemical structures of (**a**) 1,2-di-*O*-α-linolenoyl-3-*O*-β-d-galactopyranosyl-sn-glycerol and (**b**) β-sitosterol-3-*O*-β-d-glucoside isolated from chloroform fractions of methonolic extract of *A. ursinum*.

A study tested the antiplatelet effects of aqueous, chloroform and methanolic extracts of leaves of *A. ursinum* as well as the ethyl acetate fractions of the ethanolic extract (prepared by liquid-liquid extraction) in human PRP upon activation with different agonists. The ethanolic extract (10 mg/mL) showed a significant reduction in 20 µM ADP- induced aggregation by around 66%. However, the inhibitory effects against A23178 (4 µg/mL) and epinephrine (20 µM) were not significant. In addition, chloroform and ethyl acetate fractions of the methanolic extract, at a concentration of 5 mg/mL, inhibited ADP-induced platelet aggregation by almost 98% [53]. Similarly, the ethanolic extract of *A. ursinum* leaves, essential oil, ethyl acetate and chloroform factions showed significant inhibitory effects, specifically against 20 µM ADP-induced human PRP aggregation. In addition, isolated compounds from chloroform fractions such as 1,2-di-*O*-α-linolenoyl-3-*O*-β-d-galactopyranosyl-sn-glycerol (Figure 5a) and β-sitosterol-3-*O*-β-d-glucoside (Figure 5b) showed inhibitory effects on ADP-induced human PRP aggregation [64].

### 2.4. Cruciferous Vegetables

Cruciferous vegetables (family Brassicaceae) such as cabbage, Chinese cabbage, cauliflower, broccoli and kale are cultivated and consumed globally [65]. Various studies have reported on the protective effects of cruciferous vegetables against different types of cancers including lung, breast, gastric, bladder and prostate cancers [65,66,67,68]. Cruciferous vegetables are a good source of vitamins (including vitamin C, E and folic acid), minerals (including calcium, iron and zinc), flavonoids (mainly anthocyanins), phenolic acids (including hydroxycinnamic acid) and tannins [65]. However, most of the biological effects of cruciferous vegetables result from their organosulfur compounds (glucosinolates) that are hydrolysed into isothiocyanates and indole-3-carbinol by myrosinase present in plant cells during the process of chopping, cooking or freezing, or in human gut [65,69,70]. In addition, cruciferous vegetables significantly reduce CVDs-associated mortalities and exhibit antioxidant, hypotensive, hypolipidemic, hypoglycaemic and antiplatelet effects [70,71].

The ethyl acetate and *n*-butanol extracts of *Brassica oleracea* L. var. *capitata* (cabbage) leaves, *Brassica oleracea* var. *Italica* (broccoli) florets, *Brassica oleracea* var. *botrytis* L. (cauliflower) floral head, *Brassica rapa* subsp. *rapa* (turnip) root and *Wasabia japonica* rhizome (wasabi) were evaluated for their antiplatelet effects in human PRP using 10 µM ADP and 0.5 mM arachidonic acid (AA) as agonists (Table 2). In AA-induced PRP aggregation, the ethyl acetate extracts of *Wasabia japonica*, *Brassica oleracea* L. var. *capitata* and *Brassica rapa* subsp. *rapa* showed significant inhibitory effects by around 90%, 88% and 80%, respectively. The ethyl acetate extract of *Wasabia japonica* inhibited platelets by 60%, while the *n*-butanol extract inhibited platelets by 58% upon stimulation with ADP in platelets [72].

In addition, methanolic extracts of *Brassica oleraceae* L. var. *acephala* (kale) leaves exhibited significant reduction in P-selectin exposure in AA (250 µg/mL)-induced whole blood samples that were collected from patients who were diagnosed with pathological conditions such as obesity, hypertension, hyperglycaemia, and hyperlipidaemia. However, its effect on agonist-induced fibrinogen binding was insignificant [73].

**Figure 6:**
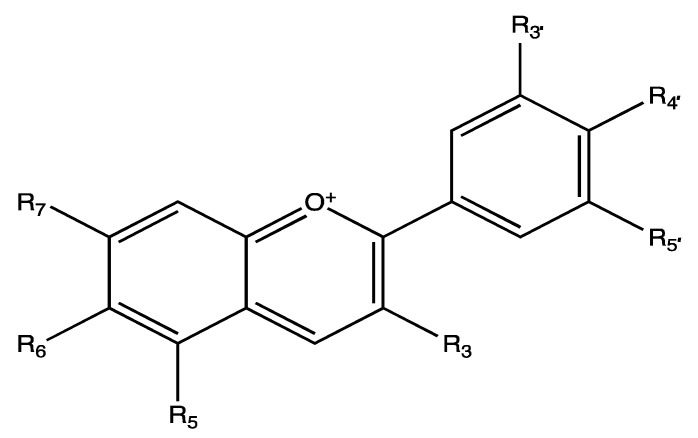
Basic structure of anthocyanins (flavylium cation).

The antiplatelet effects of the anthocyanin-rich extract were evaluated in human platelets. Anthocyanins are a group of plant pigments that belong to the flavonoid class of phytochemicals. Their basic structures consist of a flavylium cation (Figure 6) and based on the substitutions at C3, C5–C7and C3′–C5′ positions, their structures are different in diverse anthocyanins [74]. Various studies reported the health benefits of anthocyanins including antioxidant, anti-inflammatory, anti-cancer, anti-bacterial, anti-thrombotic and neuroprotective effects [74,75]. *Brassica oleracea* var. *capitata* F. *rubra* (red cabbage) leaves, which are abundant in anthocyanins (around 322 mg of anthocyanins/100 g of fresh weight), were prepared as a methanolic extract. The anthocyanin-rich extract at 5, 10 and 15 µM (calculated based on absorption coefficient) showed a significant inhibition on human isolated platelet aggregation induced by 0.5 U/mL thrombin to around 66%, 53% and 38%, respectively [76]. This extract also exhibited an inhibitory effect on lipid peroxidation in platelets which leads to inappropriate platelet activation [76,77]. At a concentration of 15 µM, it significantly reduced the production of superoxide anion in human isolated platelets activated by thrombin (6 U/mL) by around 80%, while at the concentration of 10 µM, it significantly inhibited the metabolism of AA and subsequently the formation of TXA_2_ [75,76]. At 10 µM, it significantly suppressed lipid peroxidation in platelets activated by lipopolysaccharides (LPS) from *Escherichia coli* and *Pseudomonas aeruginosa* (0.15 and 1.5 µg/mL) by around 50% (with *E. coli* LPS) and 60% (*P. aeruginosa* LPS) [77].

**Figure 7:**
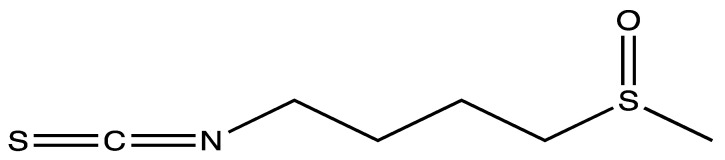
Structure of sulforaphane, an abundant sulphur compound in cruciferous vegetables.

Sulforaphane (Figure 7) is a sulphur compound that is abundant in *Brassica oleracea* var. *Italica* (broccoli) florets (62.64–982.36 µg/g of dry weight) and stem (18.11 to 274.00 µg/g of dry weight) [78] and in *Brassica oleracea* var. *capitata* F. *rubra* (red cabbage) leaves (48–101.99 µg/g of dry weight) and *Brassica oleracea* L. var. *capitata* (green cabbage) leaves (7.58–540 µg/g of dry weight) [79,80]. It has been reported that sulforaphane exhibits anti-cancer, antibiotic, antioxidant, anti-inflammatory, hypotensive and hypoglycaemic effects [69].

The effect of sulforaphane on platelet aggregation has been evaluated in previous studies [81,82]. In human isolated platelets, 25 µM and 50 µM sulforaphane markedly inhibited 1 µg/mL collagen-induced platelet aggregation by around 60% and 90%, respectively. However, the inhibitory effects of sulforaphane (25–200 µM) in platelets induced by 60 µM AA, 1 µM U46619 or 0.05 U/mL thrombin were not significantly different from controls. Moreover, 25 µM and 50 µM sulforaphane markedly inhibited the phosphorylation of PLCγ2 in platelets (Figure 2) [81]. Upon platelet activation, PLCγ2 hydrolyses membrane phospholipid, phosphatidylinositol 4,5 bisphosphate (PIP_2_) to produce the second messengers 1,4,5-trisphosphate (IP_3_), which increases the cytosolic calcium levels and diacylglycerol (DAG) to stimulate protein kinase C (PKC) which in turn activates platelet degranulation, TXA_2_ synthesis and release, and activation of integrin αIIbβ3 leading to platelet aggregation [83]. Furthermore, sulforaphane at 0.125 and 0.25 mg/kg showed a significant reduction in mortality rate due to ADP (700 mg/kg) induced acute pulmonary thrombosis in mice by 58.3% and 41.7%, respectively [81]. Similarly, the effect of 60 µM and 100 µM of sulforaphane on platelet aggregation was tested in human isolated platelets after 30 min of incubation. Upon activation with thrombin (0.1 U/mL), 100 µM sulforaphane showed 50% inhibition. However, in collagen (2.5 µg/mL)-activated platelets, both 60 µM and 100 µM of sulforaphane inhibited aggregation by nearly 100%. However, neither concentrations affected ADP (1.56 µM)-activated platelets [84]. In addition, aggregation using mouse platelets activated by 3 U/mL thrombin was significantly suppressed by sulforaphane at 10, 20 and 50 µM. In vivo tail bleeding times in mice were also prolonged to 58.2 ± 1.4 and 76.4 ± 1.2 s when 7.1 µg/mouse and 17.7 µg/mouse concentrations were used, respectively [82].

**Figure 8:**
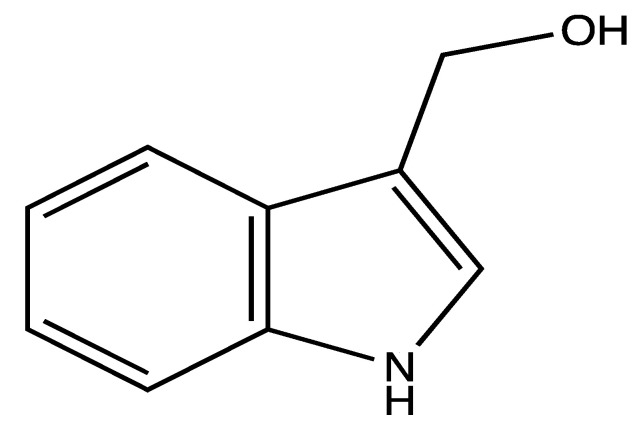
Structure of indole-3-carbinol isolated from different *Brassica* species.

Another anti-cancer compound isolated from cruciferous vegetables is indole-3-carbinol (Figure 8). This compound is produced by the hydrolysis of glucobrassicin (which is a glucosinolate) via myrosinase, during plant tissue processing, i.e., chopping, crushing, or chewing or by the human gut microflora. Glucobrassicin is a major compound in *Brassica oleracea* var. *Italica* (broccoli), *Brassica oleraceae* var. *capitata* *F. alba* (white cabbage) and *Brassica oleracea* var. *botrytis* L. (cauliflower) as it accounts for almost 50% of the total glucosinolate in those vegetables [85]. Indole-3-carbinol has been reported to possess anticancer effects, specifically against hormone-dependent cancers such as breast, uterine, ovarian and prostate [86,87].

Additionally, the antiplatelet effects of indole-3-carbinol were evaluated using various platelet functional assays. Different concentrations of indole-3-carbinol (3, 6, 12 and 25 µM) showed a significant reduction in human isolated platelet aggregation activated by 5 µg/mL collagen by around 70%. Thromboxane B_2_ (TXB_2_, a stable metabolite of TXA_2_) production was also significantly inhibited by 25 µM indole-3-carbinol by around 70%. Moreover, in a thromboembolism model in mice, the oral administration of 4.4, 8.8, 17.7 or 36.8 mg/kg of indole-3-carbinol protected mice against mortality by 67%, 70%, 70% and 89%, respectively upon stimulation with a mixture of 114 µg collagen and 13.20 µg epinephrine [88].

In another study, indole-3-carbinol (3, 6, 12 and 25 µM) displayed significant inhibitory effects on 10 µM ADP-induced human PRP aggregation by 64%, 76%, 84% and 90%, respectively. Additionally, in ADP (10 µM) activated rat PRP, oral doses of 12.5, 25, and 50 mg/kg/day of indole-3-carbinol for 14 days significantly suppressed PRP activation by 33%, 55%, 66% and 77%, respectively. The same doses significantly reduced rat brain infarction volume in a middle-cerebral artery occlusion rat model [89].

### 2.5. Green Leafy Vegetables

Green leafy vegetables are rich in flavonoids, vitamins such as vitamin A and C, glucosinolates, carotenoids, essential polyunsaturated fatty acids and nitrate [90,91]. Nitrates (NO_3_^−^) that consist of nitrogen and oxygen atoms are crucial for plant growth as they are used to synthesise amino acids and subsequently proteins. They are also important for chlorophyll formation. Plants absorb nitrates from soil through their roots and then store them in leaves [92]. Therefore, green leafy vegetables have a high content of nitrates. Nitrates display different pharmacological effects, including antioxidant, gastroprotective, antibacterial, hypotensive, and antiplatelet effects and have been found to improve endothelial function and blood flow to ischaemic tissues [92,93]. *Spinacia oleracea* (spinach) leaves have many beneficial effects on human health including antioxidant, anti-cancer, anti-inflammatory, hypolipidemic and hypoglycaemic effects [94]. Cho et al. [95,96], studied the effect of *Spinacia oleracea* (spinach) leaf extract which is a saponin-rich extract on platelet activity. Saponins are natural compounds that have shown positive impacts on cardiovascular health by acting as antioxidant, hypotensive, hypoglycaemic and hypocholesterolaemic agents [97]. Different concentrations (100, 300 and 500 µg/mL) of the *S. oleracea* saponin-rich extract showed significant inhibitory effects on aggregation in rat isolated platelets upon activation by 10 µg/mL collagen in a concentration dependent manner (53%, 50% and 40%, respectively). In addition, 500 µg/mL saponins-rich extract significantly increased cAMP and cGMP levels in platelets by around 60% compared to the controls [96].

Another leafy vegetable that demonstrated antiplatelet effects is *Eruca sativa* (Rocket) leaves [98]. 1 mg/mL concentration of 30% methanolic extract of *E. sativa* leaves was tested in human PRP, activated by 8 µM ADP, 1 mM AA and 1.5 µg/mL collagen. However, only the ADP-stimulated platelet aggregation was inhibited by around 50% (IC_50_ of 0.71 mg/mL). In addition, it inhibited P-selectin exposure from around 58% to 42% and the release of TXB_2_ in platelets. A single dose of 200 mg/kg of the extract was tested in a thrombosis model in mice and it postponed the artery occlusion time to 60 min compared to 30 min in the control group and it significantly reduced the maximum occlusion from 100% to almost 56% [98].

## 3. Fruits

### 3.1. Tomatoes

*Solanum lycopersicum* (tomatoes) is widely consumed all over the world in different forms, i.e., raw, cooked or processed. They are known for their antioxidant, anti-inflammatory, anticarcinogenic, hypoglycaemic and lipid-lowering effects due to their active components including phytosterols, flavonoids, nucleosides and carotenoids [99,100,101]. Specifically, lycopene, which accounts for more than 80% of total carotenoids in tomatoes, and β-carotene, which accounts for around 10%, are two important molecules that display biological effects in tomatoes [101].

**Figure 9:**
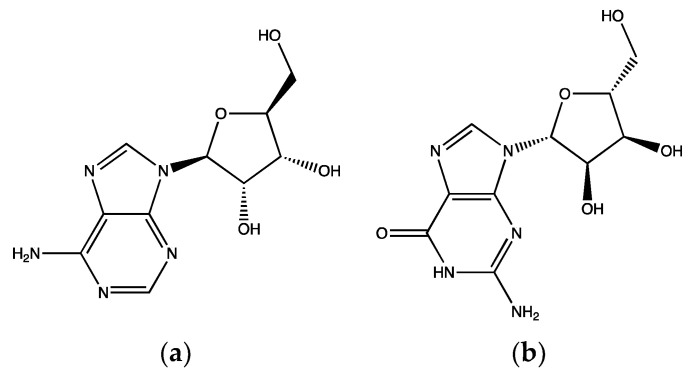
Structure of purine nucleotides isolated from *S. lycopersicum;* (**a**) adenosine and (**b**) guanosine.

Due to the beneficial effects of *S. lycopersicum* on CVD risk factors, various studies have investigated the effects of *S. lycopersicum* extracts or their isolated compounds on platelet function. Fuentes et al. [102] demonstrated the effect of methanolic extracts of *S. lycopersicum* ripe fruit and processed products (paste and pomace) and their liquid-liquid fractions (petroleum ether, ethyl acetate, and aqueous) on platelet activation. In human PRP stimulated by ADP (8 µM), 1 mg/mL of methanolic extract of ripe fruits and aqueous fractions significantly inhibited platelet aggregation by around 50%. Additionally, adenosine (Figure 9a), a purine nucleotide was isolated from the aqueous fraction of tomatoes and it significantly reduced PRP aggregation by around 45% at a concentration of 4.6 µM [103,104]. The aqueous extract (1 mg/mL) of *S. lycopersicum* pomace (industrial tomato by-product consists mainly of peels and crushed seeds) and paste (cooked and concentrated whole fruits) significantly supressed ADP-induced PRP activation by around 35–40% [102]. These data indicate that processing of *S. lycopersicum* fruits, which involves heating up to 100 °C, may not affect their antiplatelet effects.

In another similar study, the methanolic extract of *S. lycopersicum* and its petroleum ether, ethyl acetate and aqueous fractions as well as guanosine (Figure 9b) (another purine nucleotide), which was isolated from the aqueous fraction, were tested on human isolated platelets. The aqueous fraction (1 mg/mL) was found to have the highest (around 54%) inhibition on platelet aggregation, stimulated by 8 µM ADP, followed by the petroleum ether (43 ± 6%) and ethyl acetate (39 ± 8%) fractions. Moreover, 4 mM guanosine suppressed ADP-induced aggregation by 95% and ATP release by 92%. However, in collagen (1.5 µg/mL) activated PRP, aggregation was significantly inhibited by approximately 97%, with ATP release inhibited by around 72%. Guanosine also inhibited platelet spreading and sCD40L release in thrombin-activated platelets (Figure 2). Collagen-induced platelet adhesion under controlled flow was reduced by nearly 70% with 2 mM guanosine [105]. Data from the previous two studies indicate that the aqueous extract of *S. lycopersicum* is more effective as an antiplatelet agent in ADP- and collagen-activated platelets than the total extract and its fractions. This is mostly due to the purine nucleotides (adenosine and guanosine), with guanosine showing higher antiplatelet activities than adenosine.

Moreover, 40 µL aqueous extract of *S. lycopersicum* fruit significantly reduced ADP (10 µM) and collagen (2 µg/mL)-induced human PRP aggregation by approximately 70% and 41%, respectively. However, the extract did not affect platelet aggregation stimulated by 0.5 mM AA. In this study, the effects of different incubation times (5, 10, 15 and 30 min) of the extract with PRP in collagen-induced platelet aggregation were probed and the results demonstrated that the inhibitory effect of the extract (40 µL) was positively correlated with the incubation time. Platelet activation was inhibited by around 23%, 25%, 30% and 49%, respectively, for those incubation times. Furthermore, the authors suggested that the extract inhibits platelet aggregation through inhibiting phospholipase C (Figure 2), and not by increasing cAMP levels [106]. Although it was suggested that the inhibitory effects are positively correlated with the concentrations of extract tested, the precise concentrations of the extract were not stated and instead the volumes were used to express the amount of extract utilised in this study.

The effect of oral intake of *S. lycopersicum* fruit pomace on platelet function was evaluated in a human pilot, randomised, single-blind, and placebo-controlled intervention study. A total of 99 participants were divided equally into three groups. Two doses (1 g and 2.5 g) of the extract were tested against placebo and all were administered in the form of flavoured beverages for five consecutive days. The blood samples were collected on day 1 and 5 and the PRP was tested upon activation with 4 µM ADP. The inhibition (around 35%) of platelet aggregation was found to be significant, after day 5 in the group that received 1 g dose. However, the inhibitory effect of the 2.5 g dose was not found to be significant [107].

A previous study evaluated the differences in antiplatelet effects between 1 mg/mL aqueous extract of fresh *S. lycopersicum* fruit and the following commercially available processed products; sauce, ketchup, juice, and pomace in human PRP aggregation using four different agonists; 8 µM ADP, 1.5 µg/mL collagen, 1 µM AA and 30 µM thrombin receptor activator peptide 6 (TRAP-6) (Table 3). All extracts showed significant reductions in aggregation upon stimulation with ADP and collagen. In ADP activated PRP, ketchup and sauce showed the highest inhibitory effects of around 50%. In collagen activated PRP, ketchup and pomace exerted the highest inhibitory effect by around 40%. In addition, the abundant phenolic compounds in the extracts, chlorogenic, *p*-coumaric, ferulic and caffeic acids were identified and their antiplatelet effects were evaluated at a concentration of 500 µM using the same four agonists (Table 3). Generally, these compounds exhibited significant anti-aggregatory effects on ADP and collagen-stimulated platelets, but *p*-coumaric and chlorogenic acids showed the highest inhibitory effects [108]. Although the phenolic compounds showed antiplatelet effects, the toxicity of the tested (high) concentration, 500 µM was not evaluated in platelets.

**Figure 10:**
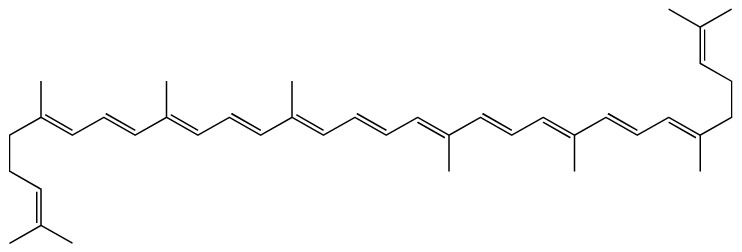
Structure of lycopene, the major constituent of *S. lycopersicum*.

In another study, the effect of different concentrations of an active compound from *S. lycopersicum*, lycopene (Figure 10) on platelet function was examined [109]. Lycopene is a natural, red pigment that belongs to carotenoid phytochemicals (specifically hydrocarbon carotenoids). The content of lycopene in fresh *S. lycopersicum* fruit is around 0.88–7.74 mg/100 g and it differs according to the stage of fruit ripeness [110,111]. It is reported that lycopene content is higher in processed products because heating facilitates the release of lycopene from plant tissues and increases its bioavailability [111]. For example, ketchup contains 9.9–13.44 mg/100 g of lycopene. The beneficial effects of lycopene were previously documented on cardiovascular health, as it acts as an antioxidant, anti-inflammatory, hypotensive, hypolipidemic and antiplatelet agent [111].

The antiplatelet effects of different concentrations (4, 6, 8, 10 and 12 µM) of lycopene were examined in human PRP stimulated by 2.5 µM ADP and 1 µg/mL collagen (Table 4). All tested concentrations of lycopene displayed significant inhibitory effects in ADP- and collagen-induced platelets. However, when compared to aspirin (140 µM), inhibitory effects of lycopene were insignificant, as aspirin reduced aggregation by 47.79% ± 15.99% and 70.37% ± 7.49% upon stimulation with ADP and collagen, respectively. In addition, the synergistic inhibitory effects of lycopene and aspirin combinations were investigated but they were not significant [109].

Furthermore, Zhang et al. [112] examined the antiplatelet effects of a pharmaceutical preparation of *S. lycopersicum*, Fruitflow^®^ powder in rat isolated platelets [113]. Fruitflow^®^ is a pharmaceutical preparation of an aqueous extract of *S. lycopersicum* fruits. It is available in a syrup and powder form and it mainly consists of active ingredients, adenosine, chlorogenic acid, rutin and lycopene all of which are reported to have antiplatelet effects [108]. At concentrations of 4 and 6 g/L Fruitflow^®^ inhibited by around 55% and 74%, respectively in 2.5 µM ADP-induced platelets. However, in collagen stimulated platelets, aggregation was reduced by 40% and 71%, respectively. Additionally, the binding of fibrinogen to integrin α_IIb_β_3_ was significantly inhibited by 32% at 6 g/L of Fruitflow^®^ in 10 µM ADP activated isolated platelets. Moreover, the antiplatelet effects of Fruitflow^®^ were tested in 2.5 µM ADP-induced isolated platelets after 4 weeks of oral administration of 25, 75 and 150 mg/kg doses of Fruitflow^®^. The 150 mg/kg dose markedly inhibited platelet aggregation to 24% [114].

### 3.2. Berries

Several studies on many varieties of berries have shown that the regular intake of berries may decrease CVD risk factors due to their antioxidant, anti-inflammatory, hypoglycaemic, and hypotensive effects. These effects have been attributed to their high content of vitamins specifically vitamin C and E, phenolic compounds mainly flavonols, phytoestrogen, minerals and essential fatty acids [115,116]. Thus, several studies have evaluated the effects of different species of berries on platelet function.

*Fragaria ananassa* (strawberry) intake has a protective effect on cardiovascular health, mainly because it has the highest antioxidant effects compared to other berries as well as other fruits and vegetables due to their high total polyphenolics and vitamin C contents [117,118]. Thus, the antiplatelet effects of *F. ananassa* were evaluated by testing the aqueous extract of fruits (0.1, 0.5, and 1 mg/mL) in human isolated platelets upon stimulation with ADP (8 µM), collagen (15 µg/mL), AA (1 mM) and TRAP-6 (30 µM). The extract inhibited platelet activation induced by AA and ADP by around 65% and 55%, respectively at a concentration of 1 mg/mL. The same concentration of extract markedly reduced the release of inflammatory markers such as sCD40L, P-selectin, chemokine ligand 5 (CCL5) and interleukin-1β (IL-1β) from platelets (Figure 2) by around 43%, 37%, 41% and 37%, respectively following stimulation with 2 U/mL thrombin. In an in vivo thrombosis model, a dose of 200 mg/kg of the extract significantly delayed thrombus formation (60 min compared to 20 min in the control group) with a maximum occlusion of 35% compared to control which resulted in 100% occlusion [119].

The antiplatelet effects of *Aronia melanocarpa* (black chokeberry fruit) were examined during hyperhomocysteinemia, which induces platelet activation. Hyperhomocysteinemia is caused by increased plasma levels of homocysteine (Hcy) (or its metabolite, homocysteine thiolactone), which is produced in the body as a result of the metabolism of methionine [120]. A high level of homocysteine is a risk factor for CVDs as it induces oxidative stress, inflammation, endothelial dysfunction and platelet aggregation. The phenolic rich extract (2.5, 5 and 10 µg/mL) of *A. melanocarpa* was combined with 100 µM homocysteine or 1 µM homocysteine thiolactone (HTL) and tested in thrombin- (0.1 U/mL) stimulated human isolated platelets (Table 5), *A. melanocarpa* extracts showed anti-aggregatory effects in a concentration dependant manner. However, the inhibitory effects of the extract were found to be insignificant on platelet adhesion to collagen and fibrinogen. In addition, when the extract was combined with Hcy or HTL, it was able to significantly reduce the stimulatory effects of both Hcy and HTL in thrombin-induced platelet aggregation and adhesion in a concentration dependant manner [121]. These effects are attributable to the antioxidant effects of *A. melanocarpa’s* phenolic rich extracts (that mainly contain flavonoids) as reported by other studies [121,122].

**Figure 11:**
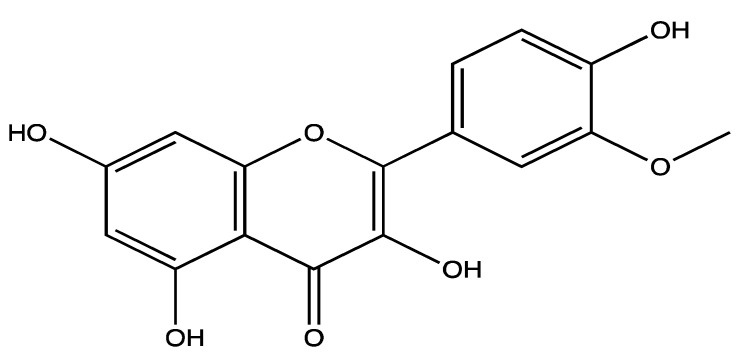
Structure of isorhamnetin isolated from *H. rhamnoides*.

In a recent human study, the effects of 5, 10 and 50 µg/mL of the phenolic fraction of 80% methanolic extract of *Hippophae rhamnoides* (sea buckthorn berry fruit) were tested on platelet adhesion to collagen and fibrinogen upon stimulation with 0.2 U/mL thrombin or 10 µM ADP activated human isolated platelets. In resting platelets, platelet adhesion was significantly reduced in a concentration-dependent manner. However, in thrombin and ADP stimulated platelets, adhesion to fibrinogen was significantly inhibited by around 65% and 55%, respectively at 50 µg/mL. In ADP activated PRP, the extract showed insignificant inhibition on aggregation [123] which indicates that the extract may have no effect on platelet ADP receptors. In a similar study, 10 µg/mL of isorhamnetin (Figure 11), an abundant flavonol in *H. rhamnoides*, and its glycoside derivative [isorhamnetin 3-*O*-beta-glucoside-7-*O*-alfa-(3′′′-isovaleryl)-rhamnoside] were tested in human PRP using 10 µM ADP, collagen 2 µg/mL and 0.1 U/mL thrombin as agonists. Isorhamnetin accounts for around 44.5–78.3% of total flavonol content in *H. rhamnoides* [124] and is reported to exert antioxidant, anti-inflammatory, anti-microbial, anti-cancer and hepatoprotective effects [125]. The antiaggregatory effects of both compounds were significant in thrombin activated platelets with around 25% effect while it was only minor in ADP and collagen activated platelets [126].

## 4. Spices

### 4.1. Turmeric

**Figure 12:**
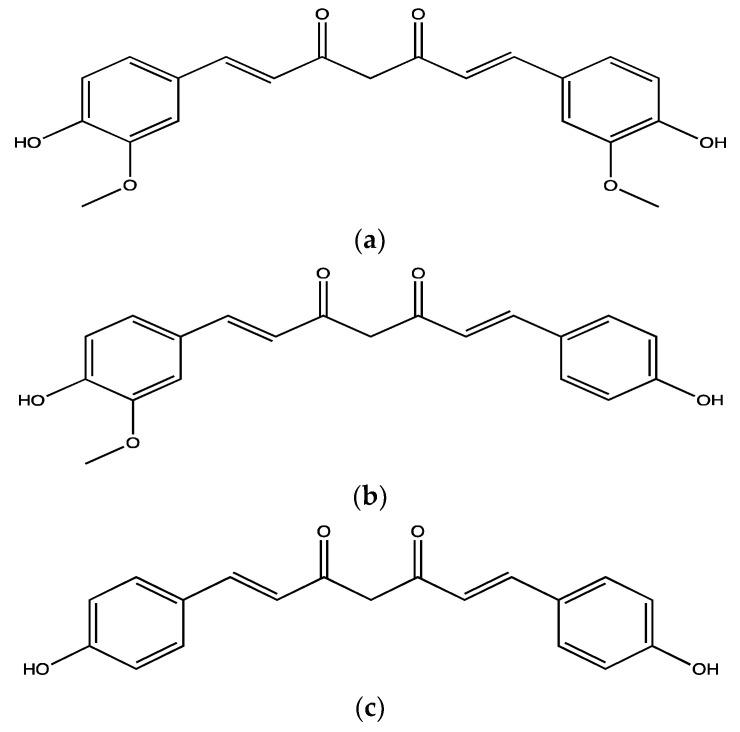
Structure of curcuminoids: (**a**) curcumin, (**b**) demethoxycurcumin; (**c**) bisdemethoxycurcumin isolated from *C. longa*.

*Curcuma longa* rhizome, which is known as turmeric, is widely used as a spice and food colouring agent, and it was routinely used in Asian traditional medicine. It was used to treat malaria, rheumatoid arthritis, hyperglycaemia and wound healing [127]. The curcuminoids such as curcumin (Figure 12a), demethoxycurcumin (Figure 12b) and bisdemethoxycurcumin (Figure 12c) are its major active constituents [128]. However, curcumin is more abundant, comprising, on average, 70–80% (*w/w*) of the total extract, whereas demethoxycurcumin accounts for 11.47–23.81% (*w/w*) and bisdemethoxycurcumin accounts for 5.97–13.88% *w/w* [129].

**Figure 13:**
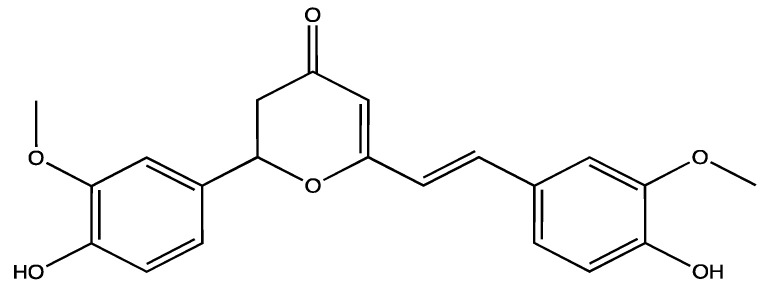
Structure of cyclocurcumin, an active constituent of *C. Longa* ethanolic extract.

A recent study showed that 250 μg/mL of ethanolic extract of *C. longa* and 25 µM of its another active compound, cyclocurcumin, (Figure 13) significantly reduced shear-stress induced platelet aggregation in a dose-dependent manner in human PRP by around 75% and 70%, respectively, with an IC_50_ value of 6.33 ± 3.29 µM for cyclocurcumin [130].

Shear-stress describes the force applied by blood flow within blood vessels which increases in blocked vessels due to thrombosis [131]. In addition, the effects of 25 µM of nine active constituents isolated from *C. longa* ethanolic extract: artumerone, bisabolatraen, bisacurone, bisdemethoxycurcumin, curcumin, 4-dehydroxybisacurone, demethoxycurcumin, β-hydroxycinnamic acid and β-sitosterol were examined (Table 6). Although these compounds reduced aggregation to different extents, they were insignificant compared to cyclocurcumin. Moreover, cyclocurcumin showed similar inhibitory effects on aggregation in human isolated platelets. Cyclocurcumin (1,5 and 10 µM) also markedly reduced intracellular Ca^+2^ levels, serotonin release, dense and α- granules secretion, fibrinogen and von Willebrand factor (vWF) binding to platelets in shear-stress-induced human isolated platelets (Figure 2) [130].

To examine the antiplatelet effects of curcuminoids, Maheswaraiah et al. [132] tested different concentrations of curcumin (10, 30 and 60 µg/mL) and the curcuminoids rich-ethanolic fraction (10, 20 and 30 µg/mL) on platelets. The curcuminoids rich fraction consists of 33% curcumin, 18% demethoxycurcumin and 48% bisdemethoxycurcumin and was tested in rat PRP aggregation stimulated by ADP (40 µM), collagen (15 µg/mL) and AA (0.75 mM) (Table 7). Generally, all tested concentrations of curcuminoids and curcumin showed reductions in aggregation to different levels when three agonists were used, but curcuminoids showed higher inhibitory effects. In addition, 20 µg/mL of curcuminoids enhanced the release of nitric oxide in rat platelets upon activation with ADP, AA and collagen.

**Figure 14:**
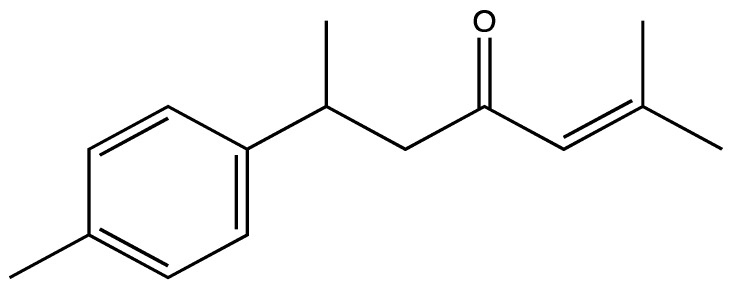
Structure of ar-turmerone, a major active component of *C. Loga* essential oil.

Another compound isolated from *C. Longa* that possesses antiplatelet effects is the sesquiterpene ketone, aromatic-turmerone or ar-turmerone (Figure 14), which is the major component of the essential oil (61.79%) and has antioxidant, anti-inflammatory, and anticancer effects [133]. In animal models, it demonstrated positive impacts on neurogenerative diseases, including Parkinson’s and epilepsy by exhibiting anticonvulsant and protective effects on neurons [134,135,136]. Antiplatelet effects of ar-turmerone were tested in rabbit isolated platelets induced by 2 µg/mL collagen at concentrations of 1, 5 and 10 µg/mL. It inhibited aggregation significantly by 60% and 80%, respectively, with an IC_50_ value of 1.2 µM [133].

In a myocardial ischemia-reperfusion injury study in rats, the effect of 500 mg/kg oral dose of *C. longa* oil on platelet function was evaluated. The oil exhibited significant improvement on neurological impairment and reduced the percentage of apoptotic cells as well as reactive oxygen species resulting from ischemic injury in rats [137,138]. The oil exhibited an inhibitory effect in rat PRP aggregation after 1 and 24 h of administration upon stimulation with 10 µg/mL collagen, 10 µM ADP and 0.64 U/mL thrombin at around 28%, 31% and 34%, respectively. However, *C. longa* oil did not affect A23187, AA and 12-phorbol 13-myristate acetate (PMA) induced platelet aggregation. In addition, oral administration of *C. longa* oil at 500 mg/kg and 1000 mg/kg markedly reduced tyrosine phosphorylation of signalling proteins with molecular weights between 55–60, 70–75, 80–85 and 90–120 kDa in rat isolated platelets upon activation with collagen, ADP, and thrombin. Furthermore, in a mouse pulmonary thromboembolism model, both doses of oil showed anti-thrombotic effects of 43 ± 7% and 63 ± 5%, respectively, after 1 h of administration compared to the 38 ± 3% obtained by 30 mg/kg aspirin. Moreover, rats treated with *C. longa* oil displayed a prolonged tail bleeding time by 18% and 25% for the 500 mg/kg and 1000 mg/kg doses, respectively compared to the controls (3–5 min) [139].

Another study reported that curcumin (1, 10, 20, 50 and 100 µM) greatly reduced platelet aggregation in aspirin pre-treated (for synergistic effects) human isolated platelets stimulated by a snake venom toxin, convulxin. Additionally, the inhibitory effects of 50 µM curcumin upon activation with 50, 100, 200, 500 ng/mL convulxin were approximately 98%, 75%, 40% and 30%, respectively. Curcumin (50 µM) also significantly suppressed platelet aggregation induced by 20 µg/mL collagen and 5 µg/mL cross-linked collagen related peptide (CRP-XL). However, it did not affect aggregation induced by a protease activated receptor 4 (PAR4) agonist, AYPGKF. Curcumin considerably suppressed the degranulation of dense granules in a concentration-dependent manner and phosphorylation of linker for activated T-cells (LAT) and PLCγ2 upon activation by 100 ng/mL convulxin (Figure 2) [140].

### 4.2. Ginger

**Figure 15:**
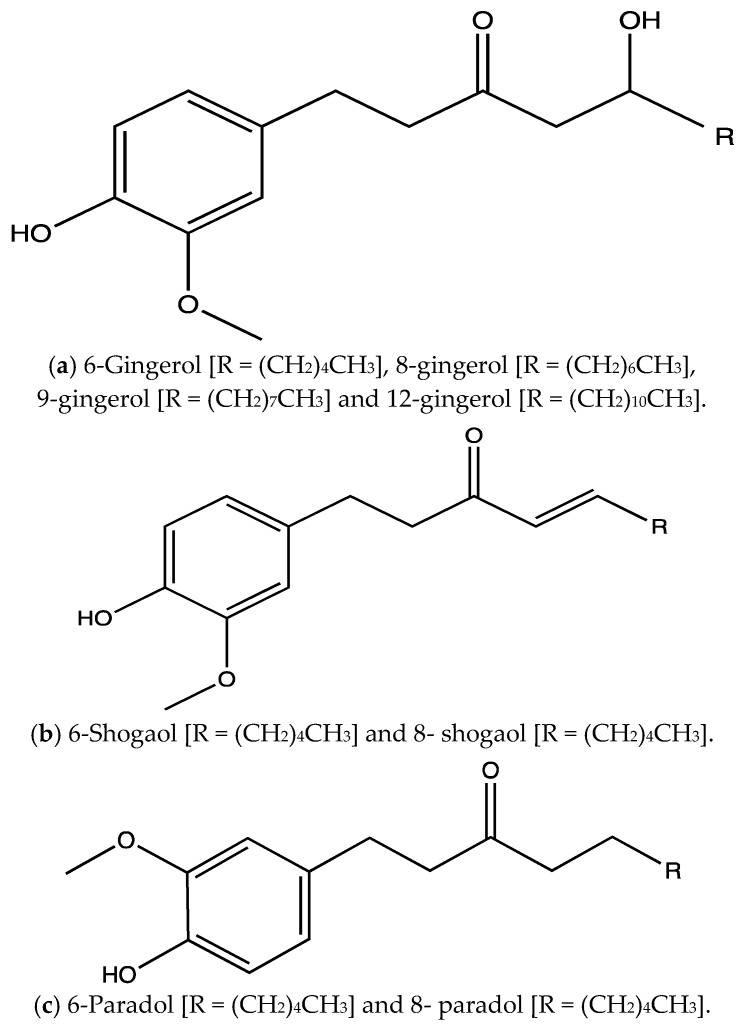
Chemical structures of different gingerols (**a**), shogaols (**b**) and paradols (**c**).

In ancient Chinese, Indian and Roman cultures *Zingiber officinale* rhizome (ginger) was widely used as a spice and for treating nausea, indigestion, diarrhoea, cough, and blood stasis [141]. Several research studies demonstrated the pharmacological effects of *Z. officinale* including, antioxidant, anti-inflammatory, anti-cancer and pain-relieving effects [141,142]. The most abundant active constituents of *Z. officinale* are gingerols, followed by shogaols and paradols. Gingerols are phenolic compounds that differ in the number of their carbon chain (after the carbonyl group) (Figure 15a). They are thermolabile compounds, as they are transformed into shogaols (Figure 15b) at high temperature (40 °C or higher) during drying or prolonged cooking [143]. After ingestion, shogaols are metabolised to paradols inside the body [144]. Thus, the most abundant gingerol in *Z. officinale* is 6-gingerol (Figure 15c) which is reported to exhibit analgesic, anti-pyretic and cytotoxic effects as well as inhibiting nitric oxide production in macrophages [142,143].

To evaluate the antiplatelet effects of *Z. officinale* powder, a placebo-controlled study examined the effect of a daily dose of 4 g of *Z. officinale* extract capsules on patients (*n* = 30) with acute myocardial infarction (more than 6 months). Patients were asked to stop their daily dose of aspirin for 2 weeks before the beginning of the study. Blood samples were collected after 45 and 90 days of administration and PRP aggregation was performed using ADP and epinephrin as agonists. Although the effect of *Z. officinale* on platelet aggregation was insignificant, a single 10 g dose led to a reduction in PRP aggregation of nearly 25% against ADP and epinephrin-induced activation after 4 h from administration. In addition, the *n*-hexane extract (5, 10, 25 and 125 µg/mL) decreased PRP aggregation induced by 0.5 µM AA in a concentration dependent manner [145].

Another study compared the antiplatelet effects of 10 µM of isolated compounds of *Z. officinale* and their synthetic analogues in 0.5 mM AA activated human whole blood aggregation (Table 8). All tested compounds showed significant antiplatelet effects. The aggregation was totally inhibited by 12-gingerol, 8-paradol, 8-shogaol, 5-hydroxy-1,7-bis(4-hydroxy-3-methoxyphenyl)-hept-6-ene-3-one and 3-hydroxy-1,7-bis(4-hydroxy-3-methoxyphenyl) heptane [146].

## 5. Edible Fungi

In the past, mushrooms were categorised within the plant kingdom and considered as vegetables. However, mushrooms are now categorised as fungi [147]. Mushrooms (also known as macrofungi) are different from microfungi (such as moulds, smuts and plant rusts) due to their visible fruiting bodies. Mushrooms lack chlorophyll, as they do not undergo photosynthesis and they do not consist of many organs such as fully developed roots, stems, leaves or flowers [148]. In recent years, there has been an increasing interest in exploring the pharmacological effects of different species of edible mushrooms due to their high mineral, vitamins, essential amino acids and fibre content along with low fat content [149,150]. Some commonly consumed mushrooms such as *Ramaria flava* (changle), *Pleurotaceae ostreatus* (oyster mushroom), *Agaricus bisporus* (button mushroom), *Lentinus edodes* (oak mushroom) and *Flammulina velutipes* (winter fungus) have demonstrated many biological effects including anti-cancer, antioxidant, antibiotic, immune enhancing, hypoglycaemic, hypocholesterolaemic, hepatoprotective and cardioprotective effects [151,152,153].

**Figure 16:**
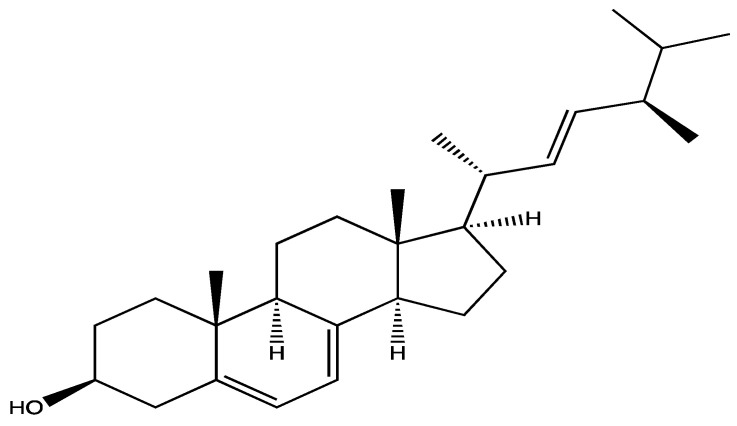
Structure of ergosterol.

Some species of edible mushrooms were demonstrated to affect platelet activation. The methanolic extract of fruit bodies of *Pleurotus florida* (500 µg/mL) was reported to significantly reduce human isolated platelets aggregation upon stimulation by 1 mM ADP by around 88% and 95% after 5- and 15 min incubation, respectively. It was suggested that this effect was due to flavonoids and polysaccharide in the extract [154]. In addition, methanolic, ethyl acetate and aqueous (5 mg/mL) extracts of *Pleurotus eous* (pink oyster mushroom) showed significant anti-aggregatory effects upon stimulation with ADP (1 mM) in human isolated platelets by 45%, 35% and 36%, respectively [155]. Poniedziałek et al. [156] studied the effect of the hot aqueous extract of eight edible mushrooms: *Agaricus bisporus*, *Auricularia auricularia-judae*, *Coprinus comatus*, *Ganoderma lucidum*, *Hericium erinaceus*, *Lentinula edodes*, *Pleurotus eryngii* and *Pleurotus ostreatus* in human whole blood platelet aggregation induced by 6.5 µM ADP. However, only *P. eryngii*, *A. bisporus*, *A. auricularia-judae* and *C. comatus*, showed significant inhibition effects, of 65.1%, 58.0%, 54.3%, and 51.6%, respectively. These effects were more significant than aspirin’s (140 µM) inhibitory effects in this study. Additionally, these four extracts, as well as the extract of *G. lucidum*, inhibited aggregation induced by 0.5 mM AA (30–34%). The inhibitory effects were linked to total polysaccharides and ergosterol (Figure 16) content and the antioxidant effects of the extracts [156].

Polysaccharides of mushrooms such as galactans, chitin and mannans are consumed as prebiotics to stimulate the growth of human gut bacteria and improve the gut health. They were also reported to have anti-tumor and immune enhancing effects [157]. Ergosterol is a sterol that found in mushroom cell membrane, and it is used as a vitamin D precursor in vitamin D supplement preparations and known to have anti-inflammatory and anti-tumor activities [158].

**Figure 17:**
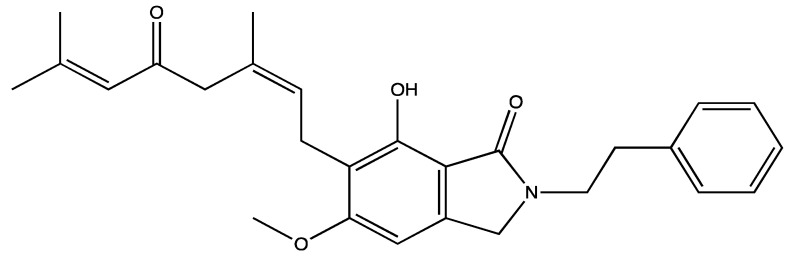
Structure of hericenone B isolated from ethanolic extract of *Hericium erinaceus*.

Moreover, isolated compounds from the ethanolic extract of *Hericium erinaceus* (lion’s mane) mushroom; hericenone B (Figure 17) significantly inhibited 3 µg/mL collagen activated human isolated platelet aggregation with an IC_50_ value of 3 µM [159].

**Figure 18:**
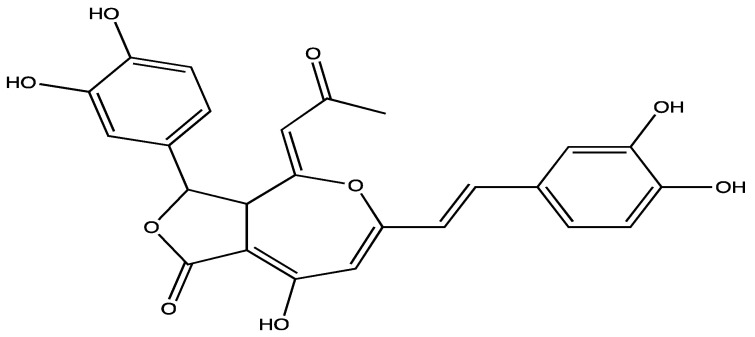
Structure of davallialactone isolated from *Inonotus xeranticus* mushroom.

In an animal study, davallialactone (Figure 18), isolated from *Inonotus xeranticus* mushroom, significantly reduced rat isolated platelet aggregation stimulated by thrombin (0.1 U/mL), collagen (2.5 μg/mL) and ADP (10 µM) in a dose-dependent manner. In addition, it suppressed the concentration of intracellular Ca^2+^ following activation with collagen. Moreover, it reduced the phosphorylation of p38 mitogen-activated kinase (MAPK) and extracellular signal-regulated kinase (ERK2) in collagen (2.5 μg/mL) and thrombin (0.1 U/mL) stimulated platelets [160].

Additionally, ethanolic extract of *Hypsizygus marmoreus* (white beech mushroom) markedly inhibited rat isolated platelet aggregation, ATP release and intracellular Ca^2+^ levels after activation with 1 μg/mL collagen, although it did not affect platelet activation by ADP (5 µM) or thrombin (0.05 U/mL) [161]. The ethanolic extract of *Cordyceps militaris* mushroom reduced rat PRP aggregation induced by 10 µM ADP and collagen 5 μg/mL in a dose-dependent manner after oral administration of 30 mg/kg, 100 mg/kg or 300 mg/kg of the extract for 3 weeks [162].

## 6. Clinical Trials for Plant Extracts and Their Isolated Compounds

Although natural products do not require Food and Drug Administration (FDA) approval to be released in the market, some pharmaceutical companies apply for FDA approval as a proof of drug’s efficacy and safety [163,164,165]. The FDA drug-review process consists of preclinical studies (on animals) followed by three phases of human studies. These phases determine the efficacy and safety of potential drug molecules using different cohorts of human volunteers. Some examples of natural plant extracts/compounds (that are already sold in the market as supplements) with antiplatelet effects that are currently in the process of FDA approval (no data released) are shown in Table 9 [166,167,168].

## 7. Conclusions

CVDs are a primary cause of deaths worldwide and they are mainly caused by impaired platelet function as well as other risk factors such as unhealthy diet, hypercholesterolemia, hyperglycaemia, hypertension, and smoking. Therefore, antiplatelet agents are predominantly used in the treatment regimen for CVD patients. Although there are various classes of antiplatelet agents that act through different mechanisms, they are associated with serious side effects and the development of resistance. Some patients with aspirin and/or clopidogrel resistance are at higher risk of recurrent strokes, large infarct size and early neurological deterioration. Some high-risk patients with recurrent ischemic stroke or transient ischemic attacks and taking three antiplatelet agents (aspirin, clopidogrel and dipyridamole) for three months experienced bleeding incidents ranging from mild to fatal although the rate of stroke recurrence was not significantly reduced. Therefore, many studies were conducted to evaluate the effects of numerous edible plants on platelet activation, with the aim of discovering novel antiplatelets agents with better bioavailability, activity and safety profiles whilst promoting the regular intake of healthy diet.

Indeed, plants are important sources for drug discovery to treat different diseases including CVDs. Several prescribed CVD medications are derived from plants, and they mainly comprise alkaloid, cardiac glycosides and polyphenolic phytochemicals. Most of the alkaloid-derived CVD drugs (such are deserpidine and reserpine) are used to treat hypertension. In addition, digoxin and digitoxin (common heart failure and antiarrhythmic drugs) are cardiac glycosides, whereas the phenolic compounds aspirin and hesperidin are used as antiplatelet drugs. However, developing new medications for CVDs is challenging because of the complicated treatment plans due to coexistence of comorbidities and other chronic conditions such as hyperlipidaemia and hypertension. These conditions increase the possibility of developing serious side effects and drug–drug interactions that might affect the bioavailability. Moreover, the complex plant extracts that contain numerous active compounds are often difficult to separate in reasonable quantities (large scale extraction and isolation are needed) and semisynthetic or synthetic strategies may be required.

Plant extracts or their active constituents can be consumed as supplements to act synergistically with prescribed medications to improve outcomes. In Chinese clinical practice, certain plant-based supplement formulations are used as adjunct treatments with CVD medications for better outcomes. For example, *Rhodiola sacra* and *Rhodiola kirilowii* capsules and injection formulations are prescribed regularly for ischemic heart disease patients (doses are adjusted according to patient’s condition) [169]. The roots and rhizomes of both species are known to act as antioxidant, cytotoxic, antidepressant and cardioprotective agents. Data from a meta-analysis study (*n* = 1672) demonstrate that using one of the *Rhodiola* formulations significantly improved ischemic heart disease symptoms (chest pressure, chest pain and shortness of breath) and electrocardiography compared to registered medications alone [169,170,171]. However, the use of plants supplements in CVDs is not always supported by clinical practice. The main concerns are that plants supplements do not need approval by the FDA or European Medicines Agency (EMA) to be released in the market. There is a lack of controlled clinical studies regarding the efficacy and safety, and insufficient knowledge of possible interactions between supplements active constituent (s) and drugs. The underreporting of adverse effects or poisoning due to consumption of plant products to the local and international regulatory organisations is another major concern. Indeed, plant supplements may be used to improve patients’ outcomes, but must be used under the guidance/supervision of health care providers. Finally, in studies that investigate the effect of plant extracts and/or phytochemicals on CVDs, human clinical trials should be considered as part of the FDA drug-approval process to determine the efficacy, safety and their interactions with other molecules.

Overall, plants are known to be a rich source for bioactive compounds that have numerous beneficial effects on cardiovascular health. They can be consumed safely as part of our diet in the form of fresh raw fruit and vegetables as recommended by health organisations. The WHO recommends the daily consumption of at least 400 g of fruit and vegetables (excluding starchy vegetables), which is equivalent to five portions daily. Based on this recommendation, many countries began a 5-a-day campaign in 1990. However, its importance has been better acknowledged since 2000, as governments have started to promote its importance in schools and big supermarkets, which are used to advertise this via their ready to eat fruit and vegetables [172].

The effects of consumption of five servings of fruit and vegetables on CVDs have been evaluated by many longitudinal studies as reviewed in this article. They reported that consuming five servings showed a significant reduction in the mortality rate of CVDs by almost 10% [173,174]. In addition, incidence of stroke was decreased regardless of sex and consumption duration compared to the group that consumed three or less servings [175,176,177]. Moreover, in hypotensive patients, the consumption of five servings for 12 weeks showed a significant improvement in blood flow through vasodilatation [178]. Furthermore, patients who were followed up for venous thromboembolism, who consumed 3–5 portions of fruit and vegetables, were at significantly lower risk of developing venous thromboembolism (around 27–53%) [179]. In addition, patients who survived stroke were at a lower risk (34%) of recurrence when this dietary plan was followed. Hence, consuming a minimum of 400g/day of fresh fruit and vegetables daily must be a part of the daily diet for children and adults, and especially for CVD patients. We believe that this article provides a plethora of information on the antiplatelet effects of selected and commonly used edible plants consumed regularly. This article aims to create awareness among members of the public and CVD patients on the necessity to adhere to a healthy diet. Moreover, it will encourage scientific communities to further investigate the impacts of these plants for the prevention and treatment of CVDs. Although we covered a wide range of commonly available edible vegetables, fruits, spices and fungi in this article, we were unable to discuss several other edible plants (e.g., green tea) that also exhibit antiplatelet effects due to their occasional and/or limited medicinal uses.

## Figures and Tables

**Table 1:** The effects of conventional oven and microwave cooking, different preparation methods and cooking time on antiplatelet activities of *A. cepa* [49].

Cooking Time (min).	Type of Processing	Effects on Platelet Aggregation
**Conventional Oven Cooking**		
10	Crushed	No inhibition or activation effects
20 & 30		Pro-aggregatory by ~25%
10	Chopped into quarters	85% inhibition of aggregation
20 & 30		Pro-aggregatory by ~40%
10 & 20	Whole (intact) bulb	85% inhibition of aggregation
30		Pro-aggregatory by ~30%
**Microwave cooking**		
2	Crushed	Insignificant inhibition
4		No inhibition or activation effects
8		Pro-aggregatory by ~25%
2	Whole (intact) bulb	Pro-aggregatory by ~20%
4		Pro-aggregatory by ~30%
8		Pro-aggregatory by ~40%

**Table 2:** Effects of extracts of selected cruciferous vegetables on human-platelet activation induced by ADP and AA [72].

		(%) Inhibition of Platelet Aggregation Induced by	
Vegetable Name	Extract	ADP	AA
*Brassica oleracea* L. var. *capitata* (cabbage)	*Ethyl acetate*	28%	88%
*Brassica oleracea* var. *Italica* (broccoli)		40%	17%
*Brassica oleracea* var. *botrytis* L. (cauliflower)		8%	10%
*Brassica rapa* subsp. *rapa* (turnip)		30%	80%
*Wasabia japonica* (wasabi)		62%	90%
*Brassica oleracea* L. var. *capitata* (cabbage)	*n-Butanol*	22%	60%
*Brassica oleracea* var. *Italica* (broccoli)		33%	0%
*Brassica oleracea* var. *botrytis* L. (cauliflower)		10%	0%
*Brassica rapa* subsp. *rapa* (turnip)		4%	5%
*Wasabia japonica* (wasabi)		58%	62%

**Table 3:** Antiplatelet effects of aqueous extracts of *S. lycopersicum* and its isolated phenolic compounds [108].

KERRYPNX		(%) Inhibition on Aggregation		
	ADP	Collagen	AA	TRAP-6
**Aqueous Extract**				
Fresh fruits	40	20	8 (ns)	3 (ns)
Sauce	48	30	7 (ns)	5 (ns)
Ketchup	50	40	6 (ns)	10 (ns)
Juice	30	28	10 (ns)	6 (ns)
Pomace	38	38	20	22
**Isolated Compounds**				
Caffeic acid	35	42	20	25
Chlorogenic acid	69	50	22	19
Ferulic acid	47	36	ns *	ns *
*p*-Coumaric acid	71	69	41	ns *

ns = not significant and * ns = not significant and not mentioned in the paper.

**Table 4:** Inhibitory effects of lycopene and its combination with aspirin on ADP- and collagen-stimulated platelets [109].

	(%) Inhibition on Aggregation	
	ADP	Collagen
**Lycopene Concentration (µM)**		
4	41.22	50.01
6	43.19	49.86
8	43.62	51.89
10	45.18	51.48
12	44.47	49.20
**Lycopene (L, µM) and Aspirin (A, µM) Combinations**		
L4 + A70	53.19	73.17
L8 + A70	43.47	66.97
L4 + A140	53.12	71.27
L8 + A140	47.71	68.49

**Table 5:** Inhibitory effects of different extracts of *A. melanocarpa* combined with Hcy (homocysteine) and HTL (homocysteine thiolactone) on platelet aggregation and adhesion [121].

	(%) Inhibition		
	Platelet Aggregation	Collagen Adhesion	Fibrinogen Adhesion
**Extract (µg/mL)**			
2.5	29.41	1.70	0.10
5	56.47	2.30	1.10
10	65.88	4.20	4.10
**Extract (µg/mL) + Hcy (100 µM)**			
2.5 + Hcy	18.40	9.00	12.00
5 + Hcy	40.50	18.90	29.50
10 + Hcy	48.90	32.30	43.80
**Extract (µg/mL) + HTL (1 µM)**			
2.5 + HTL	26.30	16.30	17.70
5 + HTL	39.40	35.00	33.80
10 + HTL	51.30	47.40	45.50

**Table 6:** Inhibitory effects of various compounds isolated from *C. longa* on platelets [130]. SIPA-shear stress induced platelet aggregation.

Isolated Compounds (µg/mL)	(%) Inhibition of SIPA
Artumerone	20
Bisabolatraen	30
Bisacurone	20
Bisdemethoxycurcumin	18
Curcumin	15
4-Dehydroxybisacurone	16
Demethoxycurcumin	5
b-Hydroxycinnamic acid	19
b-Sitosterol	22

**Table 7:** Inhibitory effects of curcuminoids and curcumin on rat PRP aggregation [132].

	(%) Inhibition		
	ADP	Collagen	AA
**Curcuminoids (µg/mL)**			
10	15	20	18
20	38	40	45
30	70	80	78
**Curcumin (µg/mL)**			
10	15	15	20
30	20	38	50
60	70	70	80

**Table 8:** Inhibitory effects of phenolic compounds of *Z. officinale* on whole blood aggregation [146].

	% Inhibition
**Isolated Compounds**	
6-Gingerol	86
8-Gingerol	96
9-Gingerol	86
12-Gingerol	100
6-Paradol	90
8-Paradol	100
6-Shogaol	91
8-Shogaol	100
**Synthetic analogues**	
3-Hydroxy-1-(4-hydroxy-3-methoxyphenyl) dec-4-ene	92
2-Hydroxy-1-(4-hydroxy-3-methoxyphenyl) dodecan-3-one	96
5-Hydroxy-1,7-bis(4-hydroxy-3-methoxyphenyl)-hept-6-ene-3-one	100
3-Hydroxy-1,7-bis(4-hydroxy-3-methoxyphenyl) heptane	100

**Table 9:** Plant extracts and/or their compounds with antiplatelet effects that are currently in clinical trials [166,167,168].

Antiplatelet Extracts	Isolated Compounds	Study Phase
Ginseng root extract: *Panax ginseng* (Korean red ginseng) *Panax notoginseng* (Chinese ginseng) *Panax quinquefolium* (*American ginseng*) *Panax japonicas* (Japanese ginseng)	Ginsenosides	Phase 3
*Ginkgo biloba* (maidenhair tree) leaves	Flavonoids (ginkgo-flavone glycosides) and terpenoids (ginkgolides and bilobalide)	Phase 3
*Gynostemma pentaphyllum* (miracle grass) leaves	Gypenoside saponins	Phase 2
*Salvia miltiorrhiza* (red sage) roots	Salvianolic acid B	Phase 2
Not defined	Berberine	Phase 3
*Colchicum autumnale* (autumn crocus)	Colchicine	Phase 3

## Data Availability

All data and appropriate references are included within this manuscript.
